# Investigation of protein quaternary structure via stoichiometry and symmetry ınformation

**DOI:** 10.1371/journal.pone.0197176

**Published:** 2018-06-04

**Authors:** Selcuk Korkmaz, Jose M. Duarte, Andreas Prlić, Dincer Goksuluk, Gokmen Zararsiz, Osman Saracbasi, Stephen K. Burley, Peter W. Rose

**Affiliations:** 1 Hacettepe University, Faculty of Medicine, Department of Biostatistics, Ankara, Turkey; 2 Trakya University, Faculty of Medicine, Department of Biostatistics and Medical Informatics, Edirne, Turkey; 3 RCSB Protein Data Bank, San Diego Supercomputer Center, University of California, San Diego, La Jolla, CA, United States of America; 4 Erciyes University, Faculty of Medicine, Department of Biostatistics, Kayseri, Turkey; 5 RCSB Protein Data Bank, Department of Chemistry and Chemical Biology, Center for Integrative Proteomics Research, Rutgers, The State University of New Jersey, Piscataway, NJ, United States of America; 6 Institute for Quantitative BioMedicine, Rutgers, The State University of New Jersey, New Brunswick, NJ, United States of America; 7 Rutgers Cancer Institute of New Jersey, Rutgers, The State University of New Jersey, New Brunswick, NJ, United States of America; 8 Skaggs School of Pharmacy and Pharmaceutical Sciences, University of California, San Diego, La Jolla, CA, United States of America; 9 Structural Bioinformatics Laboratory, San Diego Supercomputer Center, University of California, San Diego, La Jolla, CA, United States of America; University of Michigan, UNITED STATES

## Abstract

The Protein Data Bank (PDB) is the single worldwide archive of experimentally-determined three-dimensional (3D) structures of proteins and nucleic acids. As of January 2017, the PDB housed more than 125,000 structures and was growing by more than 11,000 structures annually. Since the 3D structure of a protein is vital to understand the mechanisms of biological processes, diseases, and drug design, correct oligomeric assembly information is of critical importance. Unfortunately, the biologically relevant oligomeric form of a 3D structure is not directly obtainable by X-ray crystallography, whilst in solution methods (NMR or single particle EM) it is known from the experiment. Instead, this information may be provided by the PDB Depositor as metadata coming from additional experiments, be inferred by sequence-sequence comparisons with similar proteins of known oligomeric state, or predicted using software, such as PISA (Proteins, Interfaces, Structures and Assemblies) or EPPIC (Evolutionary Protein Protein Interface Classifier). Despite significant efforts by professional PDB Biocurators during data deposition, there remain a number of structures in the archive with incorrect quaternary structure descriptions (or annotations). Further investigation is, therefore, needed to evaluate the correctness of quaternary structure annotations. In this study, we aim to identify the most probable oligomeric states for proteins represented in the PDB. Our approach evaluated the performance of four independent prediction methods, including text mining of primary publications, inference from homologous protein structures, and two computational methods (PISA and EPPIC). Aggregating predictions to give consensus results outperformed all four of the independent prediction methods, yielding 83% correct, 9% wrong, and 8% inconclusive predictions, when tested with a well-curated benchmark dataset. We have developed a freely-available web-based tool to make this approach accessible to researchers and PDB Biocurators (http://quatstruct.rcsb.org/).

## Introduction

**Fig 1:**
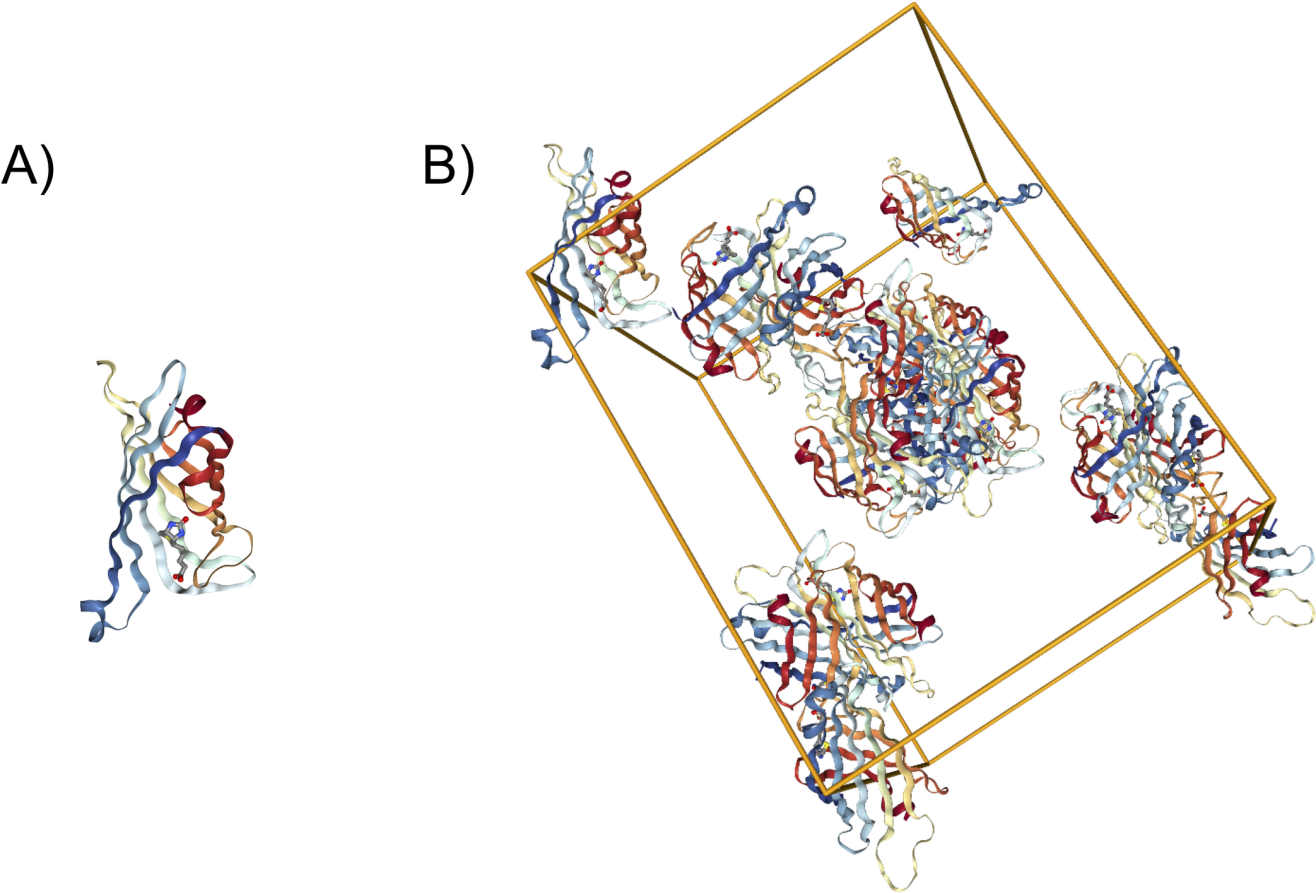
3D structure information for PDB entry 1STP. A) Monomeric asymmetric unit. B) Unit cell containing asymmetric units within the crystal lattice.

The Protein Data Bank (PDB, pdb.org) [1] provides detailed information about the three-dimensional (3D) structures of biological macromolecules, including proteins and nucleic acids. The PDB was established in 1971 with only 7 X-ray crystal structures of proteins and now contains more than 125,000 structures (as of January 2017). Today, the PDB archive is managed by the international Worldwide Protein Data Bank (wwPDB, wwpdb.org) partnership [2], which includes the RCSB Protein Data Bank (RCSB PDB, rcsb.org) [1], the Protein Data Bank in Europe (PDBe, pdbe.org), Protein Data Bank Japan (PDBj, pdbj.org), and BioMagResBank (BMRB, bmrb.org). The majority (~90%) PDB structures were determined by X-ray crystallography. This experimental method yields 3D atomic level structures of the so-called asymmetric unit (Fig 1A), which is the repeating unit that makes up the crystal (Fig 1B). Knowledge of the 3D structure of the asymmetric unit and intermolecular interactions among asymmetric units does not provide sufficient information to reveal conclusively the oligomeric structures of protein assemblies, because is often not possible to distinguish biologically relevant intermolecular contacts from contacts that merely stabilize the crystal lattice.

Many proteins form structurally well-characterized thermodynamically stable multimeric complexes, which are important for biological function [e.g., hemoglobin occurs in nature as a heterotetramer (A2B2) with a cyclic (C2) symmetry and dihedral (D2) pseudo-symmetry] [3]. Experimental methods, such as size exclusion chromatography or analytical ultracentrifugation are sometimes required to ascertain the correct oligomerization state for a protein structure determined by X-ray crystallography. Alternatively, correct oligomeric state information may be inferred by comparison with better characterized homologous proteins or be provided by the PDB Depositor as metadata. It can also be predicted using computational methods, such as PISA (Proteins, Interfaces, Structures, and Assemblies) [4] or EPPIC (Evolutionary Protein-Protein Interface Classifier) [5]. Since the PDB was established in 1971, oligomeric state information has been obtained from Depositors or predicted by PQS [6] and more recently with PISA. Although experimental evidence for the oligomeric state was not a mandatory data item in legacy PDB deposition systems, collection of experimental evidence has been improved in the new wwPDB OneDep global deposition, biocuration, and validation system [7].

Quaternary structures of protein homomers and heteromers can be characterized by two main descriptors: stoichiometry and symmetry. Stoichiometry describes the composition of the assembly in terms of subunit number and composition. There are several widely used methods for determining the stoichiometry of protein complexes, including size exclusion chromatography [8], analytical ultracentrifugation [9], and gel-electrophoresis [10, 11]. Protein assembly stoichiometry is described using a composition formula. Typically, an uppercase letter, such as A, B, C, etc., represents each type of different protein subunit in alphabetical order. (N.B.: These letters are not the same as the chain identifiers found in PDB archival entries.) The number of equivalent subunits is added as a coefficient next to each letter. For example the stoichiometry of the two-component human hemoglobin heterotetramer is represented as A2B2 (a dimer of heterodimers composed to two distinct polypeptide chains). Symmetry is another important feature of protein tertiary and quaternary structure [12] and plays a key role in understanding protein evolution and structure/function relationships [3], [12], [13], [14], [15], [16]. At the quaternary structure level, we characterize symmetry by the point group, a set of symmetry elements, whose symmetry axes go through a single point [12]. Most oligomeric protein structures (either homomeric or heteromeric) are symmetric macromolecules, which is probably a simple consequence of how subunits associate in solution without aggregating indefinitely [17] and can be classified using closed symmetry groups [18]. They are typically described as cyclic (i.e., C2, C3, C4, …), dihedral (i.e., D2, D3, D4, …), or cubic (tetrahedral, octahedral, icosahedral). Dihedral and cyclic symmetries are geometrically related: a structure with Dn symmetry can be constructed from n dimers with C2 or from two n-mers with Cn symmetry [19]. Additionally, helical symmetry is also a common open-symmetry encountered in protein structures. In a few instances, stoichiometry and symmetry are insufficient to uniquely characterize the quaternary structure of a protein assembly, where subunits form via different interfaces, while maintaining the same stoichiometry and symmetry. PDB structure 1JYE [20] is an example, where two assemblies have A2 stoichiometry and C2 symmetry, but only one of them is biologically relevant. Our investigation does not cover these special cases, however, detailed results for alternative interfaces are available from EPPIC.

**Fig 2:**
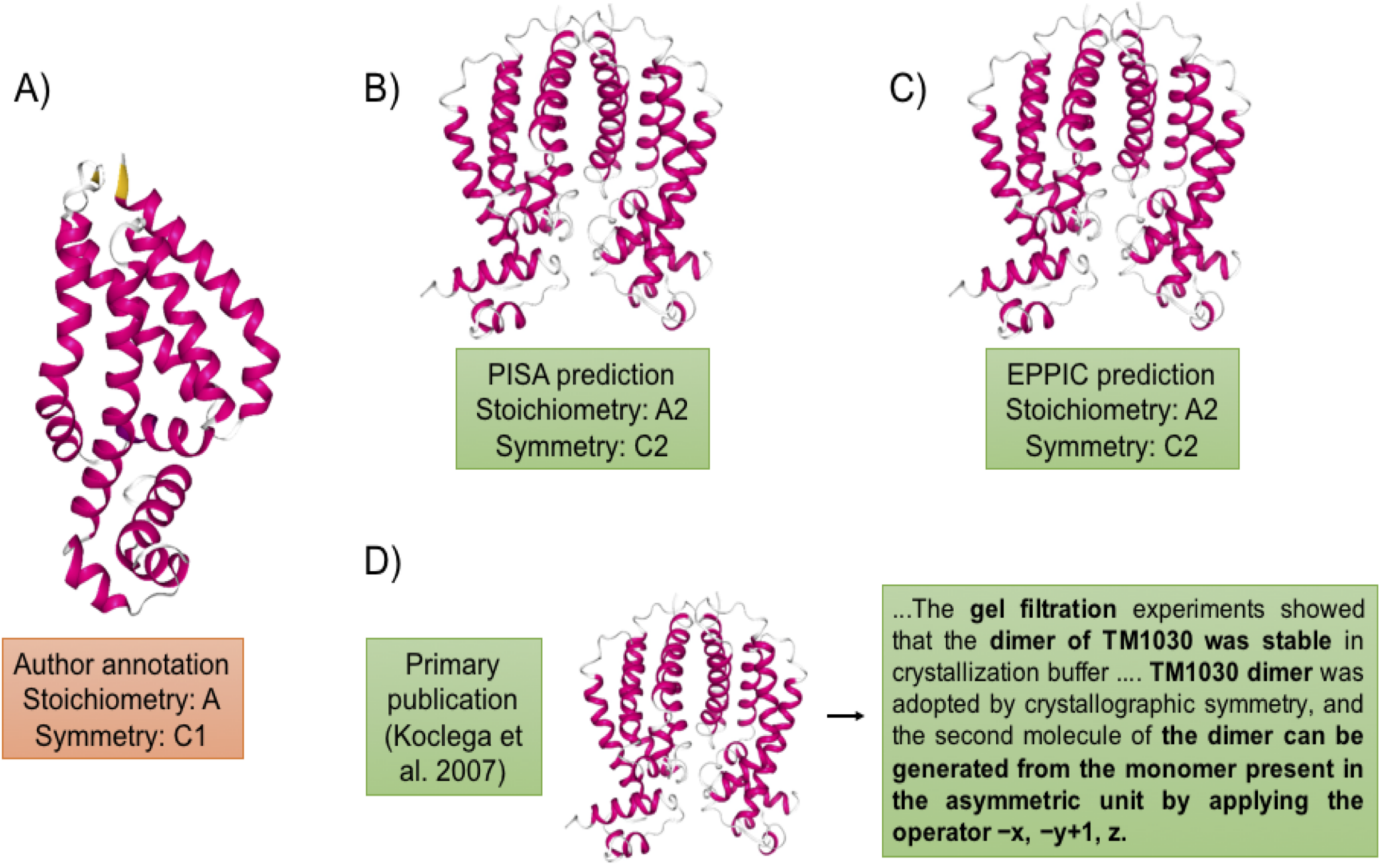
Quaternary structure annotations for PDB entry 1Z77. A) Incorrectly annotated as monomer during PDB deposition. Correctly annotated as a dimer by B) PISA, C) EPPIC, and D) the primary publication.

The PDB archive grows by more than 11,000 structures annually. However, because of incomplete data and errors made during data entry, the oligomeric state annotations provided by the PDB are not always correct and reliable [21]. In spite of great efforts to improve the quality of the PDB archive, it has been reported that there are a significant number of PDB entries with incorrect quaternary structure annotations (Fig 2). Levy put the the error rate at ~14% [22], while more recently Baskaran et al. [23] reported a lower bound for the error rate of ~7%. Development of methods for accurate detection of incorrect annotations and assignment of most probable oligomeric state is, therefore, a matter of some urgency.

This study has two main objectives: (i) to enable identification of incorrect protein quaternary structure annotations in the PDB archive, and (ii) to enable assignment the most probable quaternary structure for such cases. To accomplish these goals, we evaluated four different methods for assessing quaternary structure annotations in the PDB. First, we took an evolutionarily approach by clustering proteins related by amino acid sequence and attributed to each member of a given cluster the oligomerization state found to be most prevalent with the cluster. Second, we took a text mining approach by searching through the primary citations of indivdiual PDB entries and extracting information about oligomeric state and experimental evidence thereof. Third and fourth, we took two independent computational approaches using PISA and EPPIC, respectively, to predict oligomeric states. We aggregated results from these methods to generate a consensus prediction for the most probable oligomeric state for each protein structure in the PDB. We tested this combined approach using a well-curated benchmark dataset. During the course of this effort, we developed an efficient approach to evaluate oligomeric states of protein structures in the PDB, which we have made freely available to both PDB Biocurators and researcher as a web-based tool.

## Materials and methods

### Benchmark dataset

**Fig 3:**
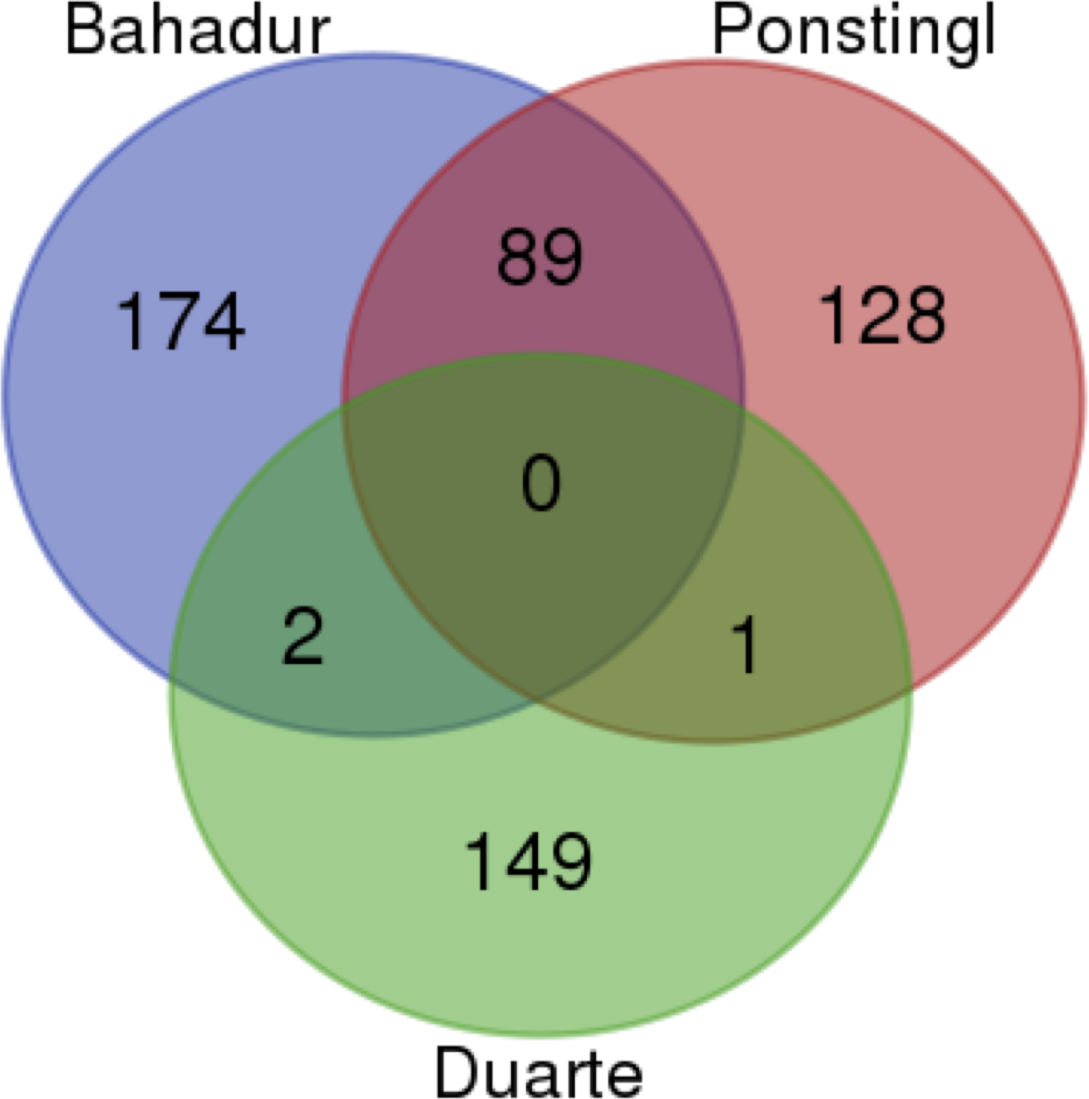
Overlapping structures between datasets. Most of the duplicates occcurred between the Ponstingl and the Bahadur datasets. These two datasets share 89 protein structures, whereas only 2 structures were found to be common between the Bahadur and the Duarte datasets, and only 1 structure was detected in both the Ponstingl and the Duarte datasets.

We aggregated three previously published, manually curated, benchmark datasets to create a considerably larger combined benchmark dataset for this work. The Ponstingl dataset [24] contains 218 protein complexes, including 55 monomers, 88 dimers, 24 trimers, 38 tetramers, and 13 hexamers. The Bahadur dataset [25, 26] contains 266 PDB entries, including 144 monomers and 122 dimers. The Duarte dataset contains [5] 152 protein structures, including 78 monomers, 62 dimers, 2 trimers, 8 tetramers, 1 hexamer, and 1 dodecamer. After removing duplicate entries (Fig 3), our final combined benchmark dataset contains 543 biological macromolecules, including 248 monomers, 209 dimers, 26 trimers, 44 tetramers, 14 hexamers, 1 octamer and 1 dodecamer.

### Sequence clustering

**Fig 4:**
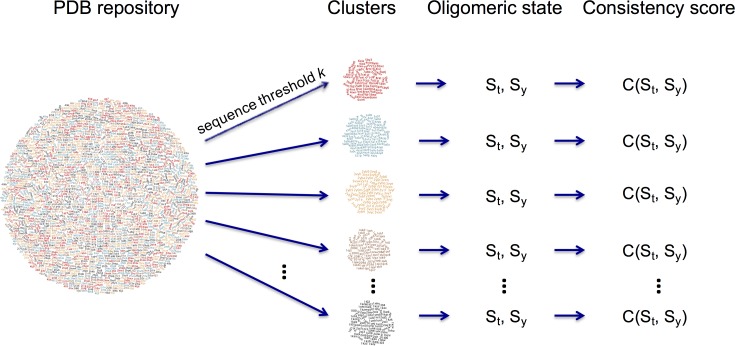
Sequence cluster construction and consistency score calculation. A consistency score is calculated based on the distribution of stoichiometry and symmetry values within each cluster. (S_t_: Stoichiometry, S_y_: Symmetry, C(S_t_, S_y_): consistency score for given S_t_ and S_y_).

Since many protein chains in the PDB are similar at the level of sequence, we use this information to cluster polypeptide chains on the basis of amino acid sequence identity and assign a representative oligomeric state for each cluster based on a consistency score. For this purpose, we first constructed sequence clusters at various identity thresholds, including 95%, 90%, 70% and 40%. Clusters were calculated using the BLASTClust algorithm [27], which detects pairwise matches with the blastp algorithm [28] and then places each sequence in a certain cluster if the sequence matches that of at least one cluster member (Fig 4).

A cluster is defined as a set of protein chains that are at least k% sequence identical to each other over 90% of the same length, and is associated with two discrete random variables:

- S_t_ represents stoichiometry and consists of a list (a,b,c,…,m) giving the number of copies of each unique molecule (e.g., A: monomer, A2: homodimer, A2B2: heterotetramer).
- S_y_ represents symmetry and takes on values such as C2 (cyclic), D4 (dihedral), H (helical).

For a certain sequence identity threshold k, a consistency score for a given stoichiometry t and symmetry y can be estimated by the joint probability of these two events:

$$C(t,y)=P(S_{t}=t,S_{y}=y)=P(S_{t}=t)\times P(S_{y}=y)$$ (1)

After consistency score calculation, we applied a binary decision rule based on the majority probability to predict a representative quaternary structure for a certain cluster. The maximum consistency score in a cluster must be greater than 0.5 to satisfy the majority rule and to predict a representative quaternary structure, otherwise the result is deemed inconclusive.

**Fig 5:**
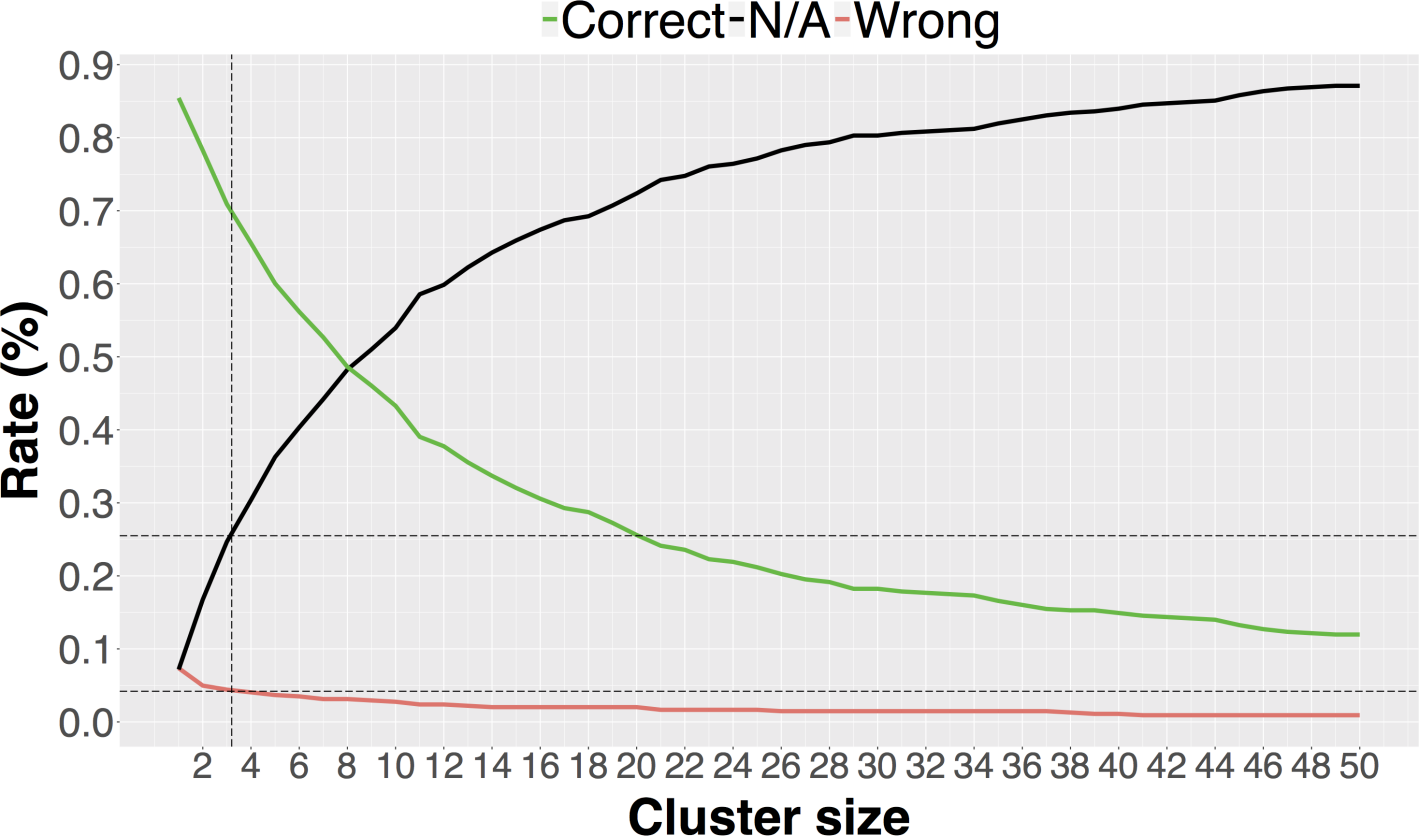
Cluster size *vs*. correct, incorrect, and N/A (not available cases) rates at 70% sequence identity.

For the consistency score to be statistically meaningful, there must be minimum number of members in a cluster. We used our benchmark dataset to determine the minimum number of cluster members for different sequence cluster identity thresholds, including 40%, 70%, 90% and 95%. For this purpose, we selected different number of cluster sizes from (n = 1,…,50) and predicted the most representative oligomeric state for each cluster using the consistency score described above. Then, we calculated the percentage of correct, incorrect, and inconclusive predictions for each minimum number of cluster members and for each sequence identity threshold. According to these results, each increment in the minimum number of cluster members reduces the number of incorrect results, but increases the number of inconclusive results. Hence, there is a trade-off between errors and cases where the predictions are not available due to an insufficient number of homologs in a cluster. In Fig 5, we plotted predictions for 70% sequence identity against cluster size. Plots for other sequence identities can be found in S1–S3 Figs. Lower sequence identities require more cluster members, while higher sequence identities require fewer. Based on our work with the benchmark dataset results, minimum cluster size should be 5 for 40% sequence identity threshold, yielding a 6% error rate and and 24% inconclusive rate. For 70%, 90%, and 95% sequence identities we found that the cluster size should be at least 3, yielding a 4% error rate and a 26% inconclusive rate. This approach was applied to both homomeric and heteromeric structures in the same way.

### Text mining

Nearly 82% of the protein structures archived in the PDB have an associated primary article as of January 2017. Using CrossRef TDM (text and data mining) services (http://tdmsupport.crossref.org/), we were able to extract information regarding oligomeric state and supporting experimental evidence from 8,600 primary publications, describing nearly 32,000 PDB entries.

First, we split the full-text article into sentences. Then, we identified sentences containing oligomeric state information using a keyword list (monomer, dimer, trimer, etc., see S1 Table for the full keyword list). Some of these sentences proved misleading or irrelevant. For example, some sentences describe the asymmetric unit not quaternary structure, and some sentences refer to protein structures other than the one of interest. We, therefore, used a machine-learning approach to eliminate non-relevant sentences by classifying sentences as quaternary structure relevant (positive) or irrelevant (negative). To train and test our machine learning algorithms for the text mining approach, we created a dataset using PDB primary papers. For this task, first, we extracted sentences from the papers using the keyword list in the S1 Table. Then, a list with experimental evidence keywords (S2 Table) was used to split these sentences into two classes. The sentences that contain experimental evidence, were used to create the positive dataset of 5500 positive sentences and the remaining sentences were used to create the negative dataset of 5500 negative sentences. Traditional machine learning algorithms require numerical inputs. Therefore, each sentence is tokenized into words. Then, we converted each word to numerical values using the term frequency-inverse document frequency (tf-idf) method [29] to create a numerical data matrix. This method reflects how important a word is to a document in a corpus using the following formula:

$$tf‑idf(n,s)=tf(n,s)\times log\left(\frac{N}{df(n)}\right)$$ (2)

were tf(n,s) represents the frequency of word n in sentence s, df(n) represents the number of sentences containing word n, and N is the total number of sentences. To improve the effectiveness of the tf-idf method, all words in each sentence were converted to lower case, with extra spaces and internal punctuation marks removed. Finally, we calculated the tf-idf score for each word in each sentence and created a data matrix for the training procedure (wherein each row represented a single sentence and each column represented a unique word). To avoid the high-dimensional data matrix, we mapped the each column (i.e. features) to a hash-table by using a hash function. In this study, we used the murmurhash3 hash function, proposed by Weinberger et al. [30]. After applying the hashing function, we used two machine-learning algorithms, support vector machines (SVM) [31] and boosted logistic regression (BLR) [32–35], to classify each sentence in a paper as positive and negative. Next, the dataset was split as 80% training and 20% test set. A grid search was used with 10-fold cross-validation to select optimal parameters in the training set. Two parameters optimized for the SVM algorithm were sigma = 0.013 and cost = 4, and the optimal number of boosting iterations found as 101 for BLR algorithm. Identical parameters were used for the test set to confirm that both datasets were on the same scale and homoscedastic relative to each other. Finally, we tested model performance on the test set (Table 1). SVM performed better than BLR in terms of accuracy, kappa, area under the ROC curve (AUC), sensitivity, negative predictive value, F1 score, and Matthews correlation coefficient. Conversely, BLR showed better specificity and positive predictive value results, suggesting that it predicts positive sentences slightly better than SVM.

**Table 1:** Test set (20% of the original data) performances of the machine learning algorithms. SVM outperforms BLR on a number of performance measures, whereas BLR has slightly better specificity and positive predictive value results.

	SVM	BLR
Accuracy	0.94	0.89
Kappa	0.87	0.78
Area Under the ROC Curve	0.98	0.92
Sensitivity	0.92	0.80
Specificity	0.95	0.99
Positive Predictive Value	0.95	0.98
Negative Predictive Value	0.93	0.83
F1 Score	0.94	0.88
Matthews Correlation Coefficient	0.88	0.81

**Fig 6:**
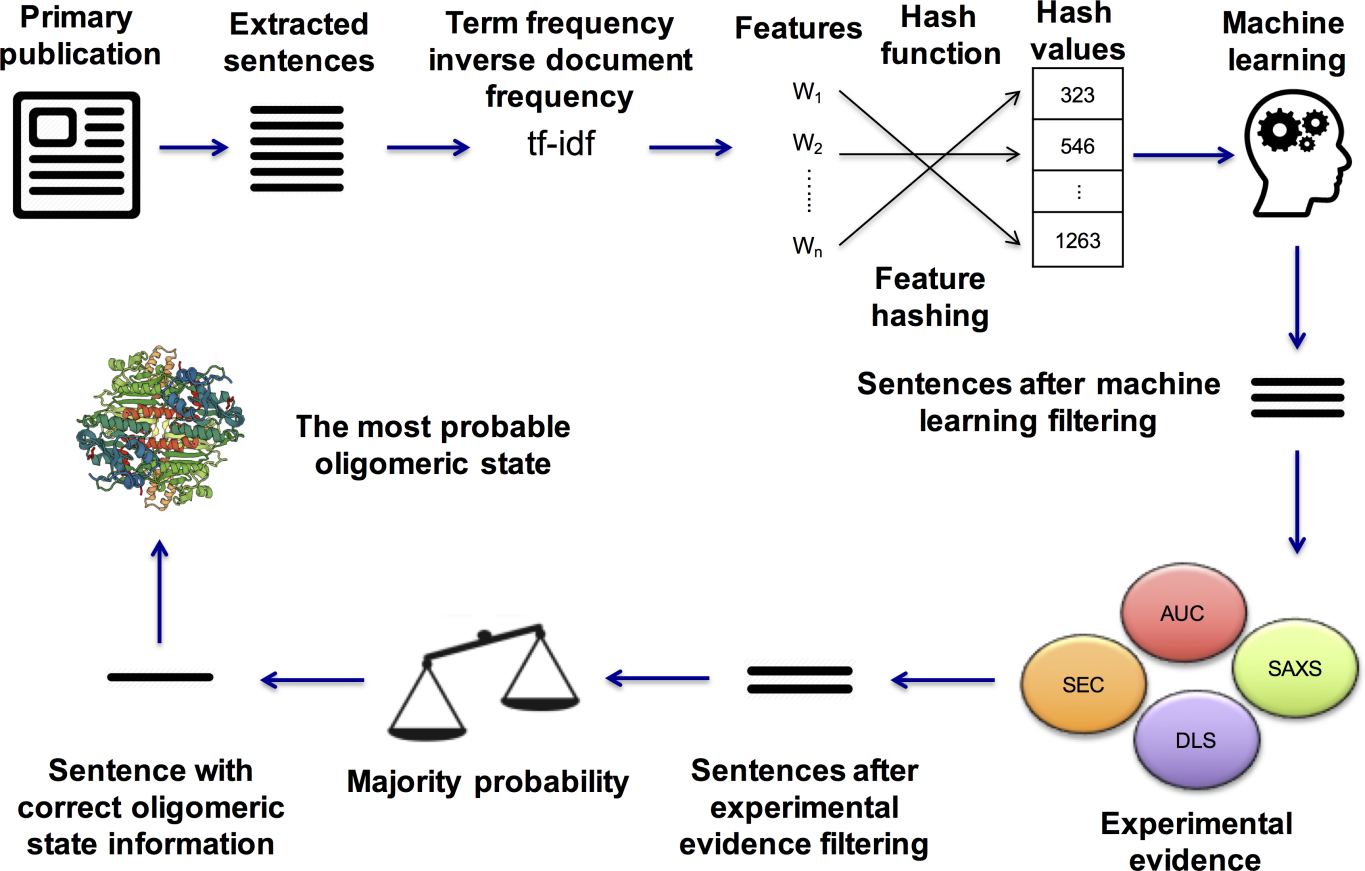
General workflow of the text mining approach. After a publication upload or download, the term frequency-inverse document frequency (tf-idf) method is used to create a numerical data matrix from words, a hash table is created using the hash function, extracted sentences are classified using machine learning alorithms, remaining sentences are searched for experimental evidence and oligomeric state information is determined by using a majority rule.

A similar approach was used to search sentences for experimental evidence of oligomeric state. An experimental evidence keyword list was used for this task (see S2 Table for the full list). After oligomeric state keyword filtering, a second filtering was applied based on these keywords. Finally, after two filtering procedures, the stoichiometry prediction for each PDB entry was made based on the majority probability of remaining oligomeric state keywords. The text mining result was deeemed inconclusive for a particular PDB entry, if machine learning algorithms failed to detect any quaternary structure related sentences and supporting experimental evidence. This approach is only used to predict stoichiometry of a protein structure. Since symmetry information is difficult to retrieve and leads to ambiguous results, we did not provide symmetry prediction using the text mining approach. A general workflow of our text mining approach can be found in Fig 6.

### PISA prediction

Following successful crystallographic structure determination efforts are made to identify biologically relevant intermolecular interactions within the crystal [36], and distinguish them from intermolcular contacts that simply stabilize the crystal lattice. The PISA program, developed by Krissinel and Henrick [4], uses a quantitative approach to address this problem [36]. The stability of an oligomeric structure is a function of free energy formation, solvation energy gain, interface area, hydrogen bonds, salt-bridges across the interface, and hydrophobic specificity [4]. PISA uses these properties to analyze protein structures and predict possible stable oligomeric states. Following successful evaluation (i.e., 90% accuracy [21]) using the Ponstingl et al. [24] benchmark data in 2007, PISA was deployed as a web server at the European Bioinformatics Institute (EBI) [36]. Soon thereafter, it was adopted as a quaternary structure validation and annotation tool for PDB archival depositions.

PISA can be accessed through a web service (http://www.ebi.ac.uk/) or as a standalone program (http://www.ccp4.ac.uk/pisa/) from Collaborative Computational Project No. 4 or CCP4 (http://www.ccp4.ac.uk/). The software provides broad information about assemblies, interfaces, and monomers, and gives information regarding possible oligomeric states, such as stoichiometry, solvent accessible surface area (ASA), buried surface area (BSA), and Gibbs free energy of dissociation score, which represents the free energy difference between the associated and dissociated states [37]. Possible oligomeric states yielding positive and negative Gibbs free energy of dissociation score are considered to be chemically stable and unstable quaternary structures, respectively [38]. Those with borderline values are deemed indeterminate.

**Fig 7:**
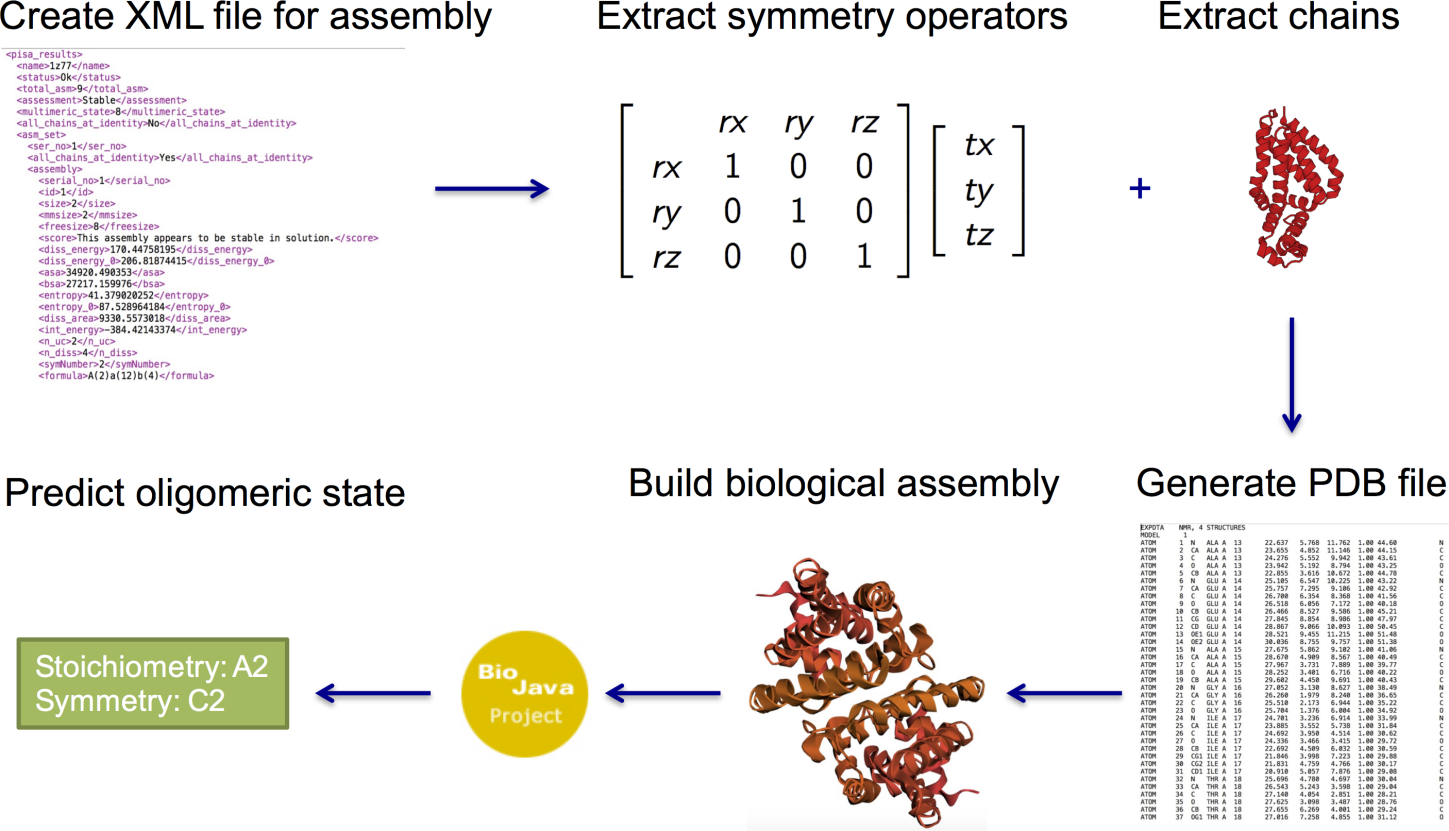
Overview of the PISA annotation procedure. An XML file is created using PISA, the file is parsed to extract symmetry operators and corresponding chains and BioJava is used to assign stoichiometry and symmetry.

In this study, we used the command line version of PISA to generate XML files for each possible oligomeric state, which include rotation/translation operators for each of the chains together with ASA, BSA, Gibbs free energy of dissociation score, entropy, internal energy, and macromolecular size. The PISA XML files were then parsed to extract rotation/translation operators and chains with which to build up the atomic coordinates of each possible oligomeric state. Finally, we used BioJava [39] to characterize each of the possible oligomeric states in terms of stoichiometry and symmetry (Fig 7).

### EPPIC prediction

To distinguish intermolecular contacts stabilizing biologically relevant oligomeric states from simple crystal contacts, an evolutionary-based classifier (EPPIC; http://www.eppic-web.org) was developed by Duarte et al. [5]. This method uses a geometric measure, number of interfacial residues, and evolutionary features to classify interfaces as biological versus crystal. Unlike other evolutionary-based methods, EPPIC uses only close homologs with >60% sequence identity to ensure homologs share high degrees of quaternary structure similarity. This approach achieved ~89% accuracy with the Ponstingl et al. [24] benchmark data. The new version of EPPIC (3.0.1) uses the pairwise interface classifications from version 2 to predict quaternary structure [40]. The crystal lattice is represented as a periodic graph from which the different valid assemblies (those with point group symmetry) can be enumerated. Based on the pairwise scores the software can then decide which of the viable assemblies is the most likely quaternary structure in solution. We obtained early access to the results from the authors. Output results for the whole PDB archive were provided in XML format, containing quaternary structure predictions for each entry in the PDB. We parsed these XML files to extract the respective stoichiometry and symmetry information for PDB entries.

### Consensus result approach

Predictions from sequence clustering, text mining, PISA, and EPPIC, were combined and a majority vote rule was applied to create a 4-method consensus result. In the following cases, predictions from individual methods were excluded:

- Sequence clustering: insufficient homologous proteins comprising the cluster (at least 3 structures for 70% sequence identity).
- Text mining: no full-text publication available or no clear information regarding quaternary structure therein.
- PISA: method does not produce any assembly result.

Moreover, in the following cases, predictions from individual methods were classified as inconclusive:

- Sequence clustering: adequate number of homologous proteins comprising the cluster but the maximum consistency score in a cluster is less than 0.5.
- Text mining: machine learning algorithm could not detect any quaternary structure related sentences which supports the experimental evidence.
- PISA: indeterminate predictions in “gray” region.
- If one of the measures, i.e. stoichiometry or symmetry, is correct and the other one is wrong.

### Web-tool development

**Fig 8:**
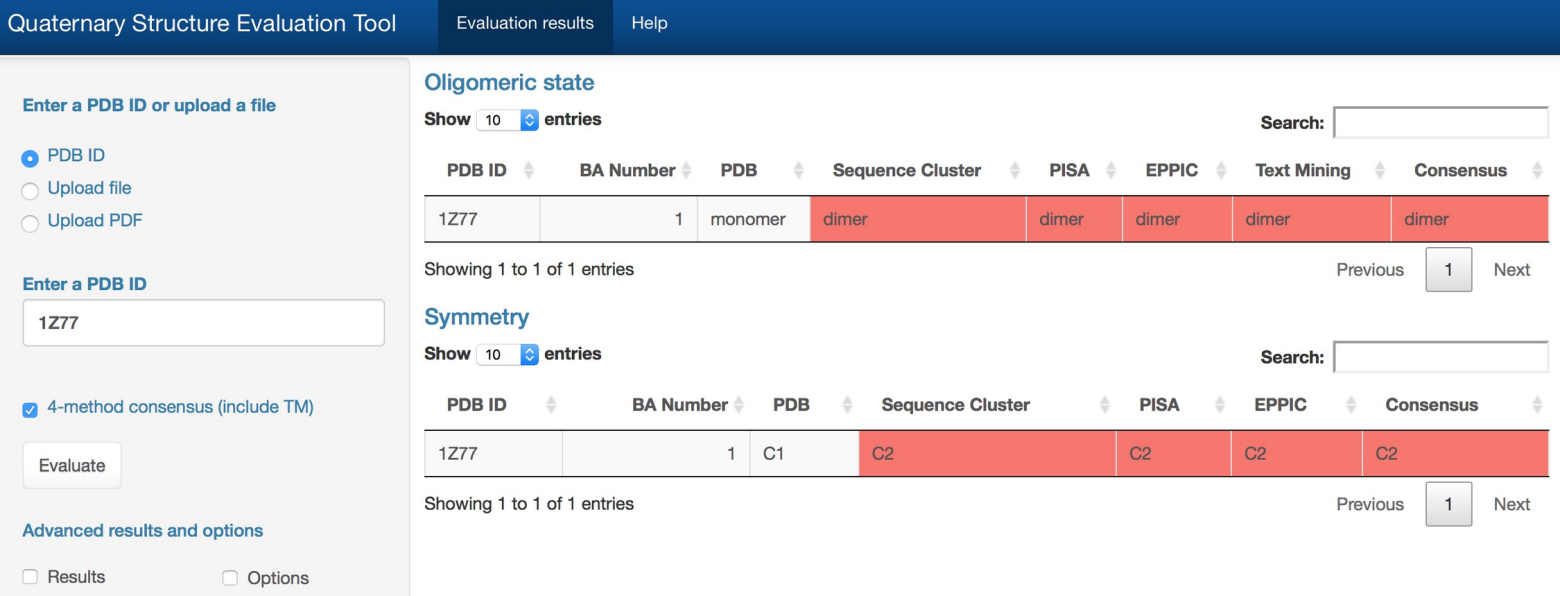
Evaluation of the quaternary structure annotation of PDB entry 1Z77. All methods (SC: sequence clustering, TM: text mining, PISA, EPPIC) are selected for the evaluation process. The output includes two parts: oligomeric state and symmetry. In the oligomeric state table, it states that 1Z77 (first column: PDB ID) has one oligomeric state prediction (second column: BA Number), and it is annotated as a monomeric structure in the PDB (third column: PDB). However, according to SC (fourth column: Sequence clustering), PISA (fifth column), EPPIC (sixth column), and TM (seventh column: Text Mining), 1Z77 is a dimeric protein. Therefore, our consensus result (eighth column) states that 1Z77 is a dimer. In the symmetry table one quaternary structure prediction is shown (second column: BA Number), having C1 symmetry (third column: PDB). However, according to SC (fourth columns: Sequence cluster), PISA (fifth column) and EPPIC (sixth column), 1Z77 has a C2 symmetry. Therefore our consensus result (seventh column) states that 1Z77 has a C2 symmetry. Red denotes divergence between current PDB annotation of oligomeric state and results provided by each of the four evaluation methods.

To make this approach accessible, we developed a user-friendly, easy-to-use web-based tool using R, JavaScript, jQuery, CSS, and HTML (Fig 8). The tool was predominantly constructed using R software [41]. Publications were downloaded either in PDF or XML format, and XML [42], Rcurl [43], tm [44], NLP [45], openNLP [46], and stringr [47] packages were used to convert PDF files to plain text files, to parse XML files, to extract sentences, and to split words. The FeatureHashing package [48] was used to map features to the hash table [30]. Machine learning algorithms were trained and tested using the caret package [49]. Data tables were built using the DT package [50], and the shiny package [51] was used to create an interactive web-based application.

The new web tool offers a wide range of methods for evaluating oligomeric states in the PDB, including

- Determination of a representative oligomeric state for a given sequence identity threshold using the sequence clustering approach with consistency scoring.
- Generate a PISA oligomeric state prediction for a given structure, rebuild the quaternary structure, and assign stoichiometry and symmetry using BioJava.
- Generate a EPPIC oligomeric state prediction for a given structure, including stoichiometry and symmetry. Predictions for multiple interfaces are included in the EPPIC predictions.
- Extract oligomeric state information with experimental evidence from any publication describing a crystal structure of a protein.

The tool has a simple user interface, requiring only four character PDB IDs as input and a single mouse click to launch the calculation. Note, in some instances, the full articles used for text mining in the benchmark may not be available in the online tool, which relies on open access PMC articles and PubMed abstracts.

## Results

### Benchmark dataset results

We applied sequence clustering, text mining, PISA, and EPPIC to our 543 structure benchmark dataset to test the performance of each approach. For the sequence clustering approach we evaluated the trade-off between the error rate and the number of non-applicable cases as a function of sequence identity. While the cluster size at 40% sequence identity (S1 Fig) is larger than at higher levels of sequence identity, quaternary structure may not be conserved at this level of sequence identity. For 70%, 90%, and 95% sequence identity, the trade-off curves are very similar for small cluster sizes (Fig 5, S2 Fig, S3 Fig) with an optimum cluster size around 3. We chose the 70% sequence identity threshold for sequence clustering because quaternary structure is generally preserved at this level of sequence identity. In comparison, EPPIC uses a 60% sequence identity cutoff. For the text mining approach, we selected the SVM classifier over BLR since it performed overall better according to the metrics in Table 1.

**Fig 9:**
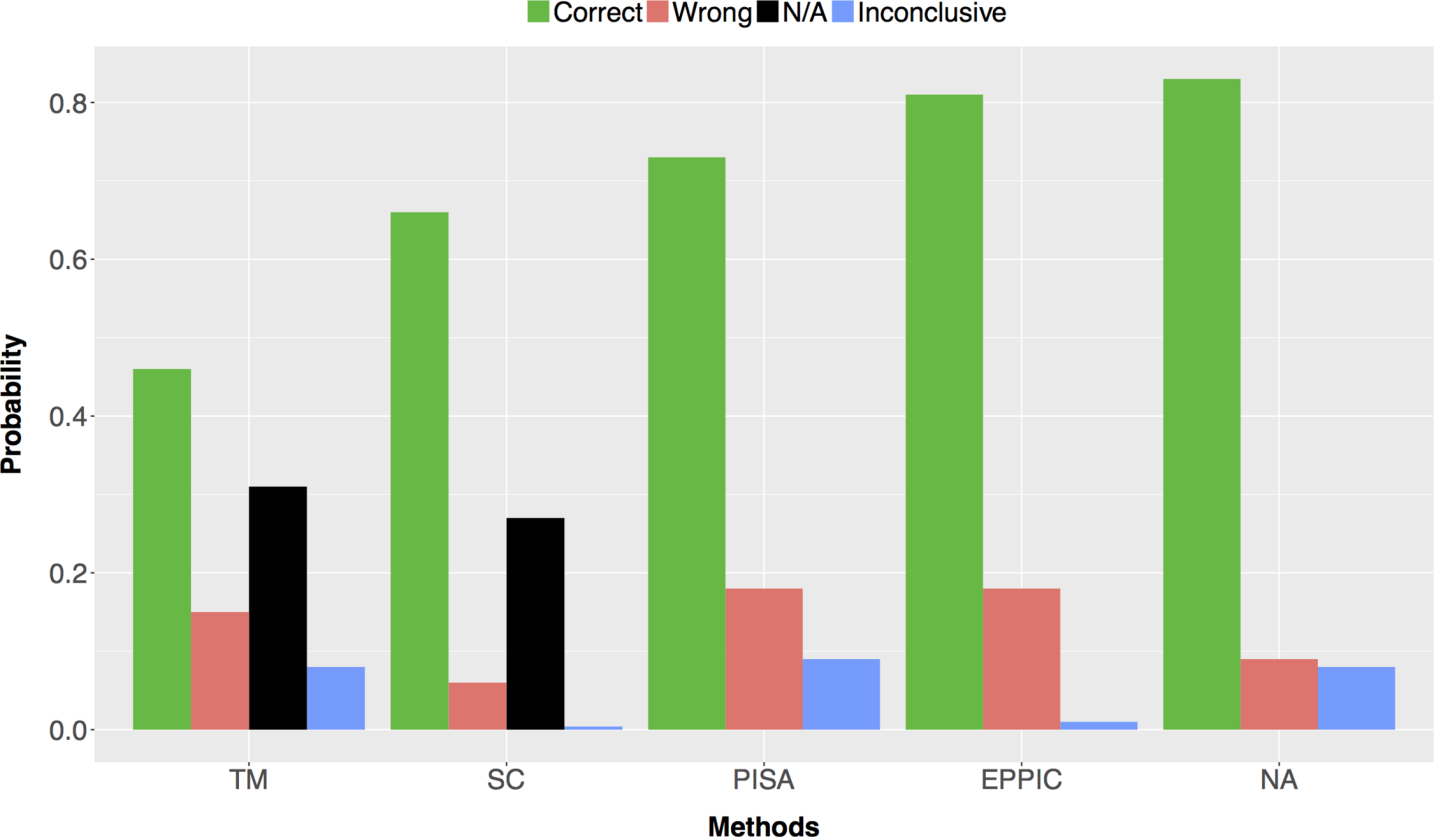
Overall performance results for the benchmark dataset using four different methods and consensus approach (N/A: not available cases).

Then, we aggregated all available results to arrive at a consensus result. S3 Table lists oligomeric state, stoichiometry, symmetry, and the individual predictions. Text mining predictions are based on stoichiometry data, whereas sequence clustering, PISA, and EPPIC use both stoichiometry and symmetry data. S4 Table summarizes the results for individual methods and the consensus predictions. Accuracy rates for individual methods ranged between 46% and 81%. We achieved 83% accuracy rate using the consensus approach (Fig 9).

**Fig 10:**
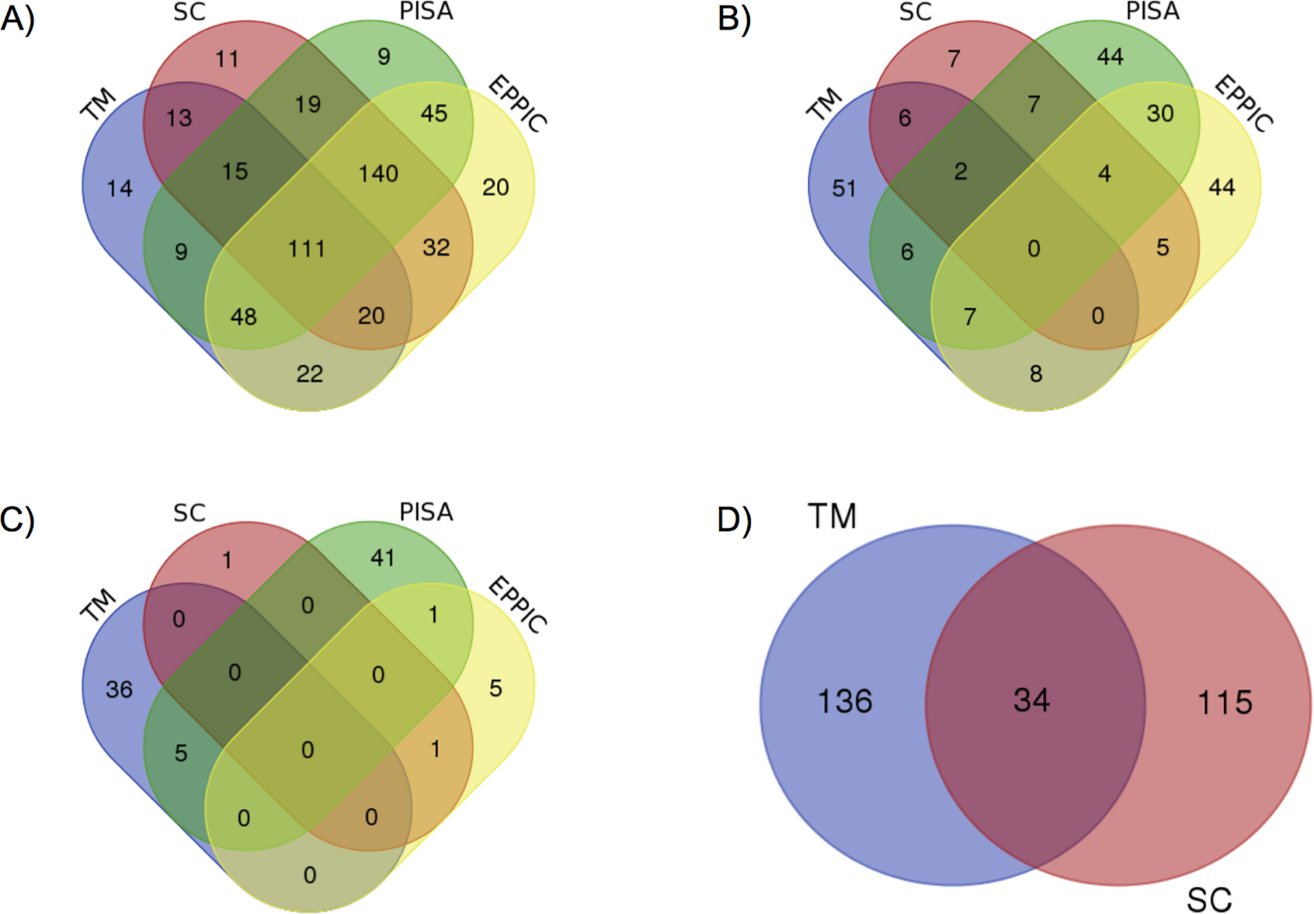
Prediction agreement of the consensus approach. A) Correct prediction agreements between methods. B) Incorrect prediction agreements between methods. C) Inconclusive prediction agreements between methods. D) Not-applicable predictions between methods.

The number of correct predictions for method-agreements are summarized in Fig 10A. The 4-method consensus correctly predicted the oligomeric states of 111 structures. The combination of SC, PISA and EPPIC (i.e. 3-method consensus) correctly predicted 140 cases. In 2-method consensus, PISA and EPPIC correctly predicted 45 cases, while SC and EPPIC correctly predicted 32 cases, and SC and PISA predicted 19 cases correctly. When we checked the number of wrong predictions (Fig 10B), there is no single structure that is incorrectly predicted by all methods. The most incorrect results occurred between PISA and EPPIC methods with 30 cases. Three methods, TM, PISA, and EPPIC, and two methods, TM and EPPIC, incorrectly predicted 7 cases. Inconclusive results (Fig 10C) mostly occurred in single methods. PISA gave 41 inconclusive results, while 36 of the TM predictions were inconclusive. Finally, the results where predictions were not-applicable (Fig 10D) occured only in TM and SC.

TM gives high inconclusive results and it can not predict stoichiometry and symmetry. Therefore, we also provide a 3-method consensus based on SC, PISA and EPPIC results. After exclusion of TM, the accuracy rate decreased to 81%, while incorrect rate increased to 11%. Thus, even though its highly inconclusive nature, TM provides useful insights regarding correct quaternary structure information.

To apply our consensus approach to a larger data set and to provide a PDB-wide result, we applied the 3-method consensus to almost 77,000 protein structures that are solved by X-ray crystallography (S5 Table). We excluded TM since it is only applicable to the subset of stuctures where the full text of the primary citation is available. According to the PDB-wide results, we detected 16.1% of the structures as possible outliers.

## Discussion

The oligomeric state (or states) of a protein represents the essential biological unit(s) that carries out a biological function in a living organism [4]. Therefore, it is crucial to determine with high reliability the biologically-relevant oligomerization state(s) of a macromolecule. This problem has been extensively studied and the scientific literature abounds with various methods and approaches. An extensive analysis of the problem can be found in Capitani et al. [21], wherein the authors reviewed main concepts of different approaches, including thermodynamic estimation of interface stability, evolutionary approaches and interface co-occurrence across different crystal forms.

The difficulty of the protein interface classification problem has led to incomplete or ambiguous experimental data, which in turn has resulted in incorrect annotations of the oligomerization states of macromolecules in the PDB. Another source of error are the simple mistakes during the deposition process. For example, sometimes authors simply identify the quaternary structure of the asymmetric unit. To the best of our knowledge, there are only two studies in the literature, which have investigated quaternary structure annotation errors in the PDB [22, 23].

In this study, we focused on two main issues: (i) detection of the incorrectly annotated biological macromolecules in the PDB and (ii) assignment of the most probable stoichiometry and symmetry annotations for those incorrect structures. We used two of the most popular and widely used methods to evaluate the protein structures through their stoichiometry and symmetry, PISA and EPPIC. To investigate author annotations, we provided a text mining approach for searching and extracting correct quaternary structure information from primary papers of the macromolecules. In addition, we utilized homology, by looking at consensus within sequence clusters, for detection of incorrectly annotated protein structures in the PDB. Finally, we obtained the results from each method, including oligomeric state and symmetry information, and calculated a consensus result for each entry in the PDB. The consensus result approach, which uses the method agreements, has improved the prediction reliability. The number of correct predictions is increased, while both the number of incorrect predictions and the number of inconclusive predictions are decreased. The best combination of the methods is found as the three methods combination of SC, PISA and EPPIC, which correctly predicted 140 cases together. On the other hand, the 4-method consensus correctly predicted 111 cases. The reason for the lower number of correct predictions in the 4-method consensus is the high rate of inconclusive and not-applicable results in the text mining approach, which is almost 40%.

The most important issue in the text mining method is the difficulty in the full-text publication access. Even though, 42% of the primary articles in the PDB are open-access publications, it is hard to get these publications using the PMC database or publishers’ websites for text mining, because these services have their own restrictions based on their respective policies. PMC does provide a service for bulk downloading of the publications for an open-access subset. However, this subset only covers 1/26 of all articles in PubMed, and that makes it impractical for text mining purposes. Some publishers, such as Elsevier and Wiley, provide their own text mining services through a specific API (application program interface). However, these services are not stable and not working all the time and more importantly they are not allowing bulk access to many papers. In addition, they do not provide all the publications through these services; instead they only provide a small subset of them. We found CrossRef TDM services as the most useful service for text mining purposes. They provide full-text links from various publishers in a user friendly and easy-to-access way. However, since some publishers only share a small part of their paper repositories, we are only able to reach 30% of the primary articles of the PDB entries.

In addition to the 4-method consensus, we also provided a 3-method consensus based on SC, PISA and EPPIC results by excluding TM. The 3-method consensus has the advantage that it can be applied to most structures in the PDB. On the other hand, TM results are useful to guide the user/biocurator to the specific papers/sentences that contain statements about the experimental evidence, as well as a resource to verify the author annotations. The performance of the methods reported here is based on a well-curated dataset. Predictions may be worse for cases outside the testing and training sets.

## Conclusion

Determination of a 3D macromolecular structure is crucial to understand the fundamental mechanisms of biological processes, such as enzymatic reactions, ligand binding, or signalling. It is also important to reveal the underlying mechanisms of diseases, such as genetic variations. Furthermore, the 3D structures of macromolecules are vital for drug design and development studies, especially in structure-based drug design. Because of these reasons, the correct quaternary structure has a critical importance.

In this study, we developed a consensus approach by aggregating predictions from three and four different methods in order to detect incorrectly annotated protein structures in the PDB through their stoichiometry and symmetry, and assign the most likely quaternary structures annotations for the possibly incorrect annotations. For this task, we first benefited from homology to cluster similar PDB entries based on a certain sequence identity threshold and to predict a representative protein structure for the cluster through a consistency score calculation. Second, we searched through the primary articles associated with the PDB entries to validate the author deposition by extracting oligomeric state and experimental evidence information using a text mining approach. Moreover, we used the PISA and EPPIC to predict the stoichiometry and symmetry of structures. Then, we combined predictions from different approaches to achieve a consensus result and to predict the most probable annotations for the possibly incorrect structures. To test the performance of our consensus approach, we created a benchmark dataset using Ponstingl et al. [24], Bahadur et al. [25], Bahadur et al. [26] and Duarte et al. [5] datasets. Our consensus approach outperformed single methods and achieved 83% correct, 9% incorrect, and 8% inconclusive predictions, respectively. Therefore, our method provides more reliable evaluation than any single approach. Rare exceptions are cases where both stoichiometry and symmetry are consistent, but subunits form alternative interfaces. In those cases, the EPPIC results list predictions for alternative assemblies. Finally, we developed a web-based tool in order to make this approach usable for researchers in the field and PDB Biocuators.

## Supporting information

S1 Fig Cluster size *vs*. correct, incorrect, and N/A (not available cases) rates at 40% sequence identity. (TIFF)

S2 Fig Cluster size *vs*. correct, incorrect, and N/A (not available cases) rates at 90% sequence identity. (TIFF)

S3 Fig Cluster size *vs*. correct, incorrect, and N/A (not available cases) rates at 95% sequence identity. (TIFF)

S1 Table Keywords list for searching quaternary structure related sentences. (DOCX)

S2 Table Keywords list for searching experimental evidence related sentences. (DOCX)

S3 Table Predictions on benchmark sets using 4 methods and consensus results. (XLSX)

S4 Table Summary of prediction results for benchmark sets. (XLSX)

S5 Table PDB-wide results. (XLSX)
