# Correction: Investigation of protein quaternary structure via stoichiometry and symmetry ınformation

**DOI:** 10.1371/journal.pone.0201403

**Published:** 2018-07-27

**Authors:** The PLOS ONE staff

In the title of this article, the dot is missing above the first letter in the word “information.” The correct title is: Investigation of protein quaternary structure via stoichiometry and symmetry information. The correct citation is: Korkmaz S, Duarte JM, Prlić A, Goksuluk D, Zararsiz G, Saracbasi O, et al. (2018) Investigation of protein quaternary structure via stoichiometry and symmetry information. PLoS ONE 13(6): e0197176. https://doi.org/10.1371/journal.pone.0197176

The second equation contains an error. The symbol between *tf* and i*df* should be a dash, not a minus sign. Please view the correct equation here:

$$tf-idf\left(n,s\right)=tf\left(n,s\right)\times log\left(\frac{N}{df\left(n\right)}\right)$$ (2)

The publisher apologizes for the errors.
