# Supplementary figures and images for: Investigation of protein quaternary structure via stoichiometry and symmetry ınformation
Source: PLoS One. 2018 Jun 4;13(6):e0197176. doi: 10.1371/journal.pone.0197176 (PMC5986128; doi:10.1371/journal.pone.0197176)

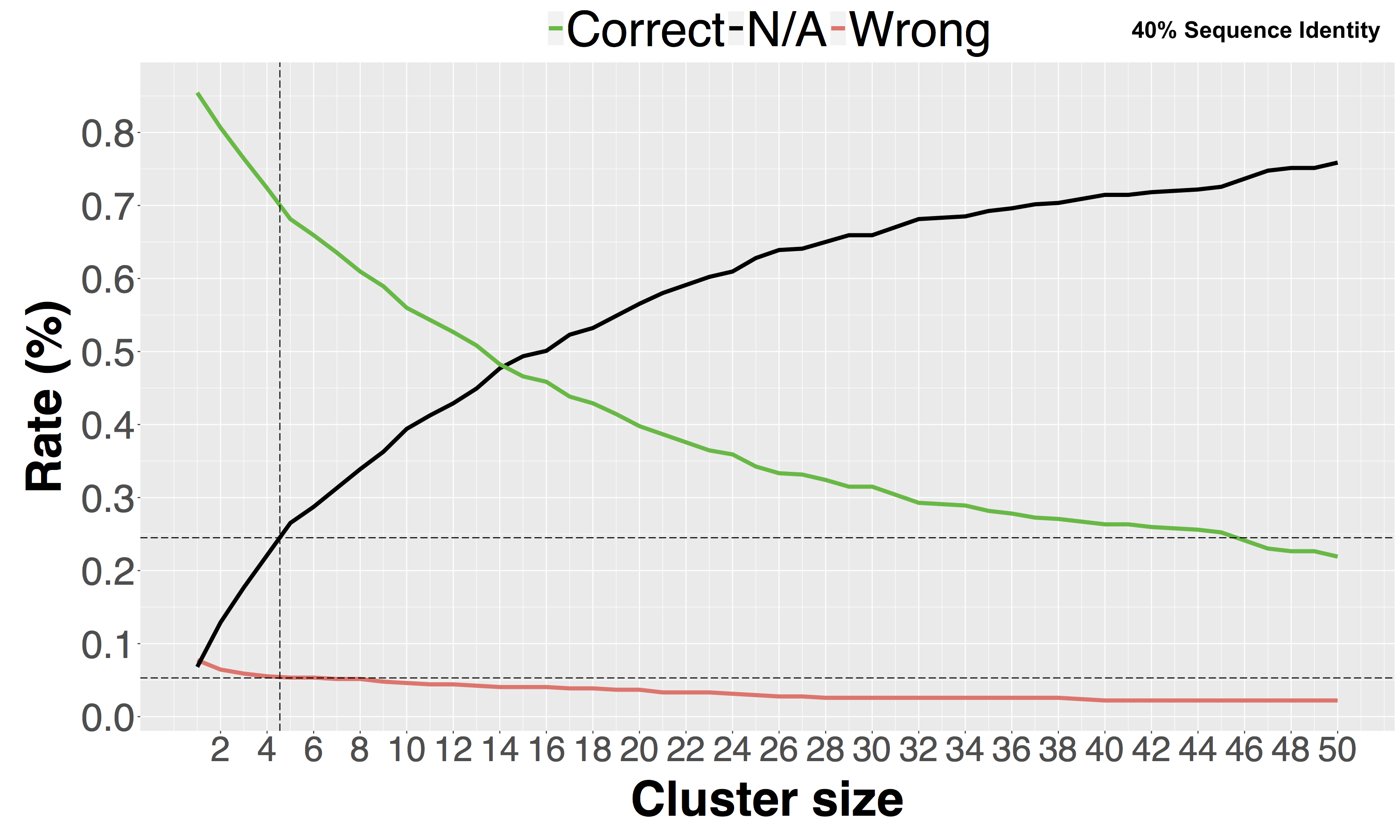

Supplement: S1 Fig — (TIFF) [file pone.0197176.s001.tiff]

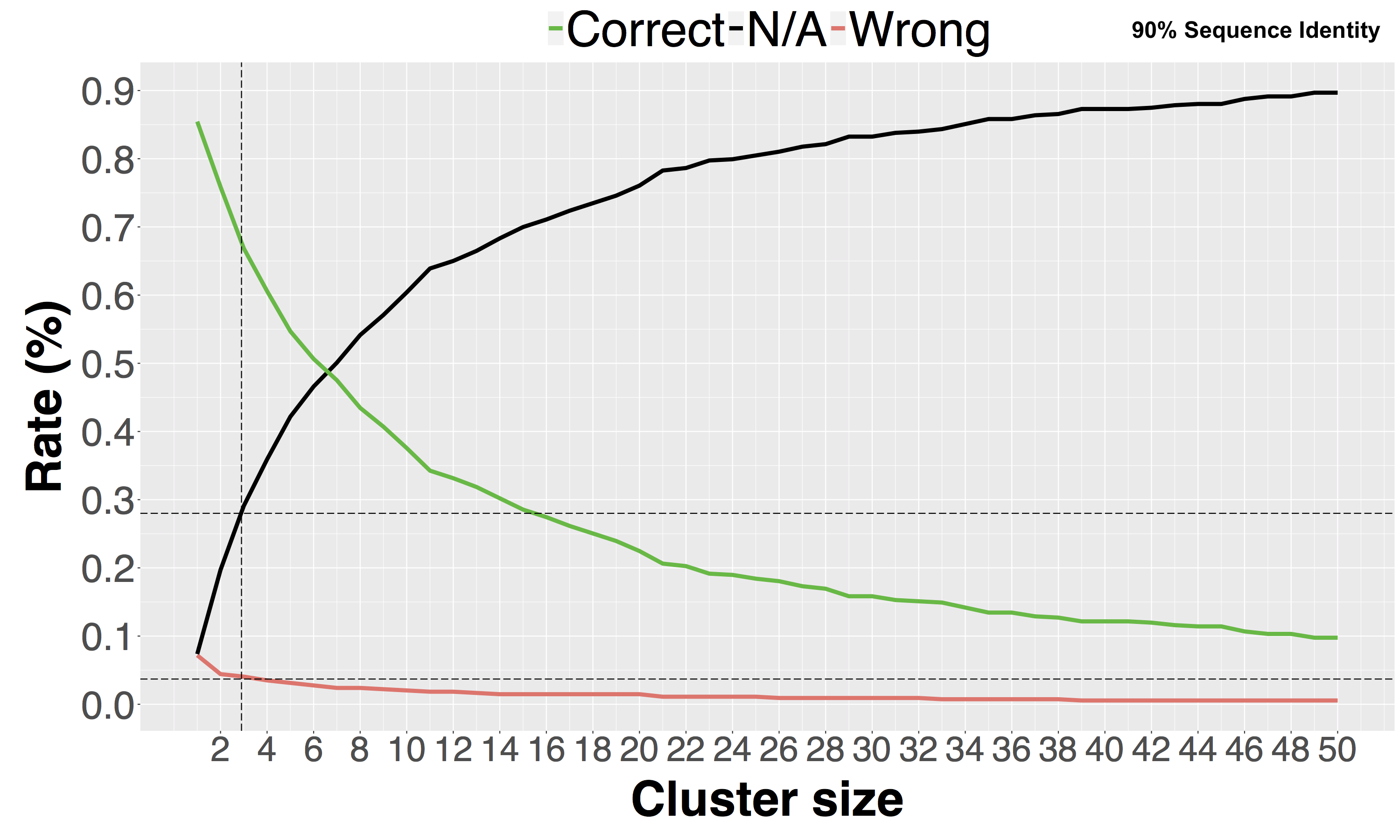

Supplement: S2 Fig — (TIFF) [file pone.0197176.s002.tiff]

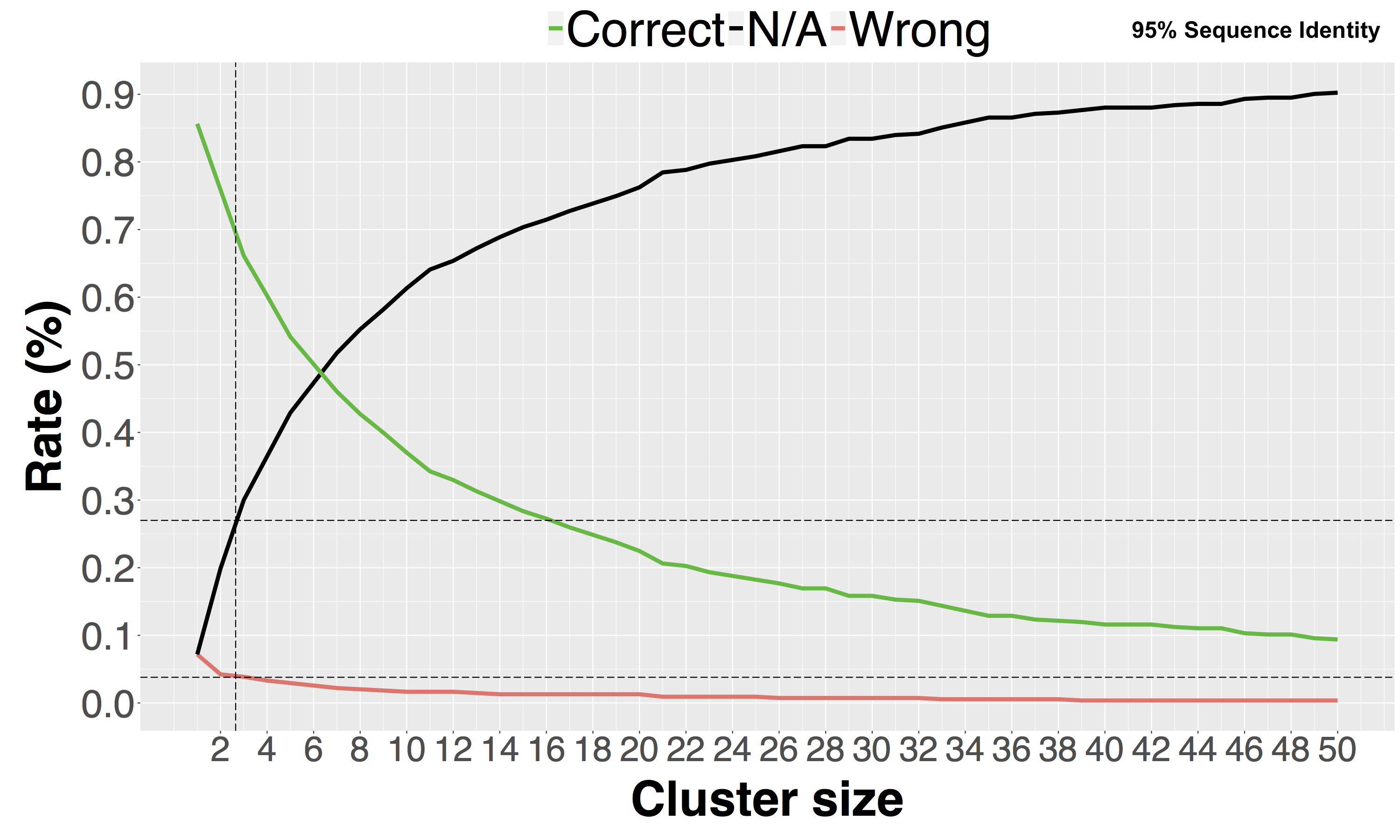

Supplement: S3 Fig — (TIFF) [file pone.0197176.s003.tiff]
