# Supplementary material for: Investigation of protein quaternary structure via stoichiometry and symmetry ınformation
Source: PLoS One. 2018 Jun 4;13(6):e0197176. doi: 10.1371/journal.pone.0197176 (PMC5986128; doi:10.1371/journal.pone.0197176)
Supplement: S1 Table — (DOCX) [file pone.0197176.s004.docx]

**S1 Table. Keywords list for searching quaternary structure related sentences.**

| "monomer" "monomeric"  "dimer" "dimeric" "homodimer" "heterodimer" "monomerofdimers"  "trimer" "trimeric" "homotrimer" "heterotrimer"  "tetramer" "tetrameric" "homotetramer" "heterotetramer" "dimerofdimers"  "pentamer" "pentameric" "homopentamer" "heteropentamer"  "hexamer" "hexameric" "homohexamer" "heterohexamer"  "trimerofdimers" "heptamer" "heptameric" "homoheptamer" "heteroheptamer"  "octamer" "octameric" "homooctamer" "heterooctamer" "tetramerofdimers"  "nonamer" "nonameric" "homononamer" "heterononamer"  "decamer" "decameric" "homodecamer" "heterodecamer" "pentamerofdimers" "undecamer" "undecameric" "homoundecamer" "heteroundecamer"  "dodecamer" "dodecameric" "homododecamer" "heterododecamer" "hexamerofdimers"  "tridecamer" "tridecameric" "homotridecamer" "heterotridecamer"  "tetradecamer" "tetradecameric" "homotetradecamer" "heterotetradecamer"  "heptamerofdimers" "pentadecamer" "pentadecameric" "homopentadecamer" "heteropentadecamer" "hexadecamer"  "hexadecamerix" "homohexadecamer" "heterohexadecamer" "octamerofdimers" "heptadecamer" "heptadecameric" "homoheptadecamer" "heteroheptadecamer" "octadecamer" "octadecameric" "homooctadecamer" "heterooctadecamer"  "nonamerofdimers"  "nonadecamer" "nonadecameric" "homononadecamer" "heterononadecamer" "eicosamer" "eicosameric" "homoeicosamer" "heteroeicosamer" "decamerofdimers" "undecamerofdimers" "dodecamerofdimers" "tridecamerofdimers"  "21mer” "22mer" "23mer" "24mer" "25mer" "26mer" "27mer" "28mer" "29mer" "30mer" |
| --- |
