# Supplementary material for: Investigation of protein quaternary structure via stoichiometry and symmetry ınformation
Source: PLoS One. 2018 Jun 4;13(6):e0197176. doi: 10.1371/journal.pone.0197176 (PMC5986128; doi:10.1371/journal.pone.0197176)
Supplement: S2 Table — (DOCX) [file pone.0197176.s005.docx]

**S2 Table. Keywords list for searching experimental evidence related sentences.**

| "size-exclusion chromatography"  "gel filtration"  "affinity chromatography"  "electrophoresis"  "gel electrophoresis"  "Native PAGE"  "active site titration"  "electron microscopy"  "mutagenesis"  "inhibition kinetics"  "titration  "analytical centrifugation"  "analytical ultracentrifuge"  "analytical ultracentrifugation"  "static light scattering"  "light-scattering"  "dynamic light scattering"  "sedimentation equilibrium"  "Small-Angle X-Ray Scattering"  "Small-angle X-Ray Scattering"  "nondenaturing gel"  "elusion profiles"  "SEC"  "AUC"  "SLS"  "DLS"  "CCL"  "FRET"  "DSC"  "SV"  "NMR"  "MALS"  "LS"  "CL"  "EM"  "UDP"  "SAXS" |
| --- |
